# Breastfeeding skills training for health care professionals: A systematic review

**DOI:** 10.1016/j.heliyon.2022.e11747

**Published:** 2022-11-18

**Authors:** Helen Mulcahy, Lloyd Frank Philpott, Michelle O'Driscoll, Róisín Bradley, Patricia Leahy-Warren

**Affiliations:** a University College Cork, Ireland; b School of Nursing and Midwifery, University College Cork, Ireland

**Keywords:** Breast feeding, Professional practice gap, Breastfeeding education, Training, Evidence-based support, Breastfeeding skills

## Abstract

**Background:**

Breastfeeding is a public health issue and the response to the low rates in the Global North needs to be multi-faceted. Within this context healthcare professionals have an important role to play in the overall multi-dimensional promotion and support of breastfeeding. As a learned skill, there is a fundamental need to improve breastfeeding skills amongst healthcare professionals.

**Aim:**

To identify, analyse and evaluate studies on breastfeeding skills education for health care professionals.

**Methods:**

The review was conducted and reported according to the Preferred Reporting Items for Systematic Reviews and Meta-Analysis (PRISMA) guidelines. Studies from June 2006 to July 2021 that examined the provision of breastfeeding skills-based education for qualified or student healthcare professionals were included. A narrative synthesis was conducted, and risk of bias independently assessed by two reviewers.

**Findings:**

Of 5,497 papers originally identified, 11 were included in the review. Nine studies were interventional, whilst two were observational. Participants included paediatric residents, midwives, nurses, care co-ordinators and other health care staff. Training took place in classrooms, practical workshops, or clinical settings. Observational or experiential teaching components in combination with theoretical knowledge were found to produce better outcomes than classroom-based interventions. However, the findings need to be interpreted with caution due to the risk of bias regarding study design-specific criteria.

**Discussion:**

There is both a paucity of studies, and from those available, a lack of quality in terms of educational interventions specifically offering skills-based training to healthcare professionals. Breastfeeding education needs to incorporate practical breastfeeding skills not just theoretical training. Lack of standardisation currently exists across guiding frameworks, course content, educator qualification and assessment strategies which impedes the optimisation of breastfeeding education and subsequent support for mothers. Serious or high risk of bias was identified in all but one of the studies included in the review.

**Conclusion:**

There is a need for high quality research evidence to optimise the design and delivery of skills-based breastfeeding education for healthcare professionals. This would have the potential to contribute to the broad suite of interventions necessary to improve support for breastfeeding.

## 1 Introduction

Worldwide, few infants are breastfed in line with evidence-based recommendations and a universal approach to supporting breastfeeding families is not established (Victora et al., 2016). As a result, global rates of breastfeeding remain lower than what is required to protect the health of women and children.

With the intention of creating environments where breastfeeding families feel supported the United Nations International Children's Emergency Fund (UNICEF) and the World Health Organisation (WHO) developed the ‘Ten steps to successful breastfeeding’ as a key component of the Baby Friendly Hospital Initiative (BFHI). Step Two recommends ‘that staff have sufficient knowledge, competence and skills to support breastfeeding’ (WHO, 2018, p. 42). However, the extent to which this education and training is implemented is unknown and particularly worrying as suggested by Gavine et al. (2016).

The two-part Lancet breastfeeding series published in 2016 (Rollins et al., 2016a; Victora et al., 2016), proposed six action points to ensure successful protection, promotion, and support of breastfeeding. These actions include the need for increased dissemination of knowledge relating to the benefits of breastfeeding; the scaling up of breastfeeding interventions to support breastfeeding skills; and the fostering of positive attitudes across all facets of society.

Although it is widely recognised that healthcare professionals are well placed to provide breastfeeding support to women and their families on an individual level, the reality of today's healthcare systems means that many healthcare professionals are affected by organisational constraints which prevent them from providing effective breastfeeding support (Biggs et al., 2018; Brown et al., 2011).

Healthcare professionals' knowledge of evidence based breastfeeding guidelines and skills alone will not be enough to change practice (Atchan et al., 2014; Dykes, 2006). Instead, educational programmes which are introduced to address breastfeeding, need to be integrated alongside designated funding and changes to organisational culture (Atchan et al., 2014; Schmied et al., 2011; United Nations Children's Fund (UNICEF), World Health Organization, 2021; World Health Organization&UNICEF 2017). Schmied et al. (2011) emphasises the need for organisational systems and services to depart institutionalised models of postnatal care, and instead adapt to models, which facilitate continuity of caregiver through midwifery led care or peer support. In doing so, the aim is to harbour relational based support and person-centred communication skills amongst healthcare workers, to deliver effective and consistent breastfeeding counselling to families (Atchan et al., 2014).

Studies globally have found that breastfeeding support from trained healthcare professionals, in conjunction with organisational support for breastfeeding are important variables for breastfeeding success (Gavine et al., 2016; Hannula et al., 2008; Rollins et al., 2016b; Henderson and Redshaw, 2011).

Despite the growing awareness surrounding the benefits of breastfeeding and the need for evidence-based breastfeeding practices, research has found that healthcare professionals continue to lack the knowledge and skills to support infant feeding effectively (Brodribb et al., 2010; Holtzman and Usherwood, 2018; Sigman-Grant and Kim, 2016). Perhaps a reason for this, is that levels of breastfeeding education varies amongst healthcare professionals and is often dependent on the chosen curriculum and mandatory trainings set out by universities and local hospitals (Weddig et al., 2011). The UNICEF &WHO (2021) acknowledge the educational challenges but make specific recommendations to assist curriculum planners in meeting needs of various personnel Lack of standardised breastfeeding education can lead to a lack of common approach, coordination, and cooperation amongst healthcare professionals, which can result in women receiving incorrect, inaccurate or/and inconsistent advice (Cross-Barnet et al., 2012; Nolan et al., 2015). Furthermore, in facilities where post registration breastfeeding education for healthcare professionals is made available, healthcare professionals who have a more direct and frequent role with breastfeeding women are likely to be the main focus (e.g., midwives and lactation consultants), with medical doctors, such as GPs and Paediatricians receiving very little formal training in relation to breastfeeding. This leads to families receiving different information depending on which professional they encounter. Thus, providing comprehensive evidence to support future breastfeeding curricula is vital for population health.

### 1.1 Study aim

The purpose of this systematic review is to identify and synthesise the evidence in relation to breastfeeding skills education for healthcare professionals.

## 2 Methods

A systematic review was conducted and reported using PRISMA 2020 statement (Page et al., 2021). A review protocol was developed to guide the review but was not registered. The Health Services Executive (HSE) commissioned the original scoping review but were not involved in the design or preparation of this manuscript.

### 2.1 Search strategy

Electronic databases Medline, CINAHL, Social Sciences and SocINDEX were searched to identify studies potentially eligible for inclusion based on pre-determined criteria. Using the PICOs framework, studies for inclusion were designs of any type and published in English between 2006 and 2020. An updated search conducted in June 2021, identified no new papers for inclusion. No geographic boundaries were applied. Studies that researched the provision of skills-based breastfeeding education to healthcare professionals were included. Studies which only included knowledge-based breastfeeding education with no skills-based components were excluded. Studies were excluded if they reported the provision of breastfeeding education to non-healthcare professionals. The reference lists of all papers that met the inclusion criteria were scanned to identify further relevant studies. The search strategy included the Boolean terms “OR”/“AND,” Medical Subject Headings (MeSH), CINAHL headings and truncation “∗”. Keywords and their synonyms were combined (see Table 1.)

**Table 1:** PICOs framework applied to the review.

PICOs framework guiding selection criteria	
**Population:**	TI (“Lactation Consultant∗” OR Midwi∗ OR “Practice Nurse∗” OR “Public Health Nurse∗” OR “Community Nurse∗” OR “Community Doctor∗” OR “General Practitioner∗” OR Physician∗ OR Medic∗ OR Paediat∗ OR Pediat∗ OR “health visitor∗” OR “health professional∗” OR “healthcare practitioner∗”OR AB (“Lactation Consultant∗” OR Midwi∗ OR “Practice Nurse∗” OR “Public Health Nurse∗” OR “Community Nurse∗” OR “Community Doctor∗” OR “General Practitioner∗” OR Physician∗ OR Medic∗ OR Paediat∗ OR Pediat∗ OR “health visitor∗” OR “health professional∗” OR “healthcare practitioner∗”
**Interventions: Comparators:**	TI (Program∗ OR Educat∗ OR Curricul∗ OR Train∗ OR Knowledge∗ OR Skill∗ OR learn∗ OR Instruct∗ OR Information OR Tuition OR Intervention) OR AB (Program∗ OR Educat∗ OR Curricul∗ OR Train∗ OR Knowledge∗ OR Skill∗ OR learn∗ OR Instruction OR Information OR Tuition OR Intervention) Healthcare professionals who do not receive breastfeeding skills training.
**Outcomes:**	TI (Breastfeed∗ OR “breast-feed∗” OR “breast fed” OR breast-fed OR Lacta∗) OR AB (Breastfeed∗ OR “breast-feed∗” OR “breast fed” OR breast-fed OR Lacta∗)
**Studies:**	Breastfeeding education programmes for healthcare professionals which include breastfeeding skills within the curriculum.
	Randomized controlled studies, quasi-experimental studies, descriptive, correlation, survey, longitudinal and case study
	Publication date 2006–2020. Updated in June 2021
	Available in the English language.

### 2.2 Study selection

Records yielded from the electronic search were exported to Mendeley Referencing Manager and after duplicates were deleted the authors paired to independently screen the titles and abstracts of the remaining papers. Potentially relevant full texts were divided in two and allocated to paired authors (HM & RB; PL-W & LP). The authors in each pair independently read the full text papers and decided whether to include or not. If a discrepancy existed between authors, the paper would then be given to the other group of paired authors to review, and a final decision made by consensus.

### 2.3 Data extraction and analysis

From the papers which met the inclusion criteria, categories were developed to be used as a framework for data extraction in the preliminary stage of analysis. These were: first author's name; country; year of publication; study design and aim; setting and sample; prerequisite training; educational curriculum (including guiding framework; content; teaching strategy; and qualifications of educator); assessment of learning; and training outcome (Table 2). One author (RB) extracted the relevant data from the chosen studies, and missing information was left blank. Data extraction was independently crosschecked by two co-authors (PL-W, HM). Discrepancies were resolved by consensus. A narrative synthesis was conducted due to the diversity of study aims, designs and findings.

**Table 2:** Data extraction from the included studies (n = 11).

Author(s), Year & Country	Design and Aim(s)	Sample & Setting	Prerequisite training	Educational curriculum				Assessment of learning	Training outcome
				Guiding framework	Content	Teaching Strategy	Educator qualification		
Bassichetto et al. (2008) Brazil	**Design:** Randomised Intervention Study. **Aim:** To evaluate the effectiveness of a young child feeding counselling course for transforming the knowledge, attitudes and practices of paediatricians and nutritionists.	Paediatricians (n = 33) and nutritionists (n = 23) involved in caring for children up to 24 months of age.	All qualified HCP's (64.3% trained >21 yrs).	The WHO Infant and Young Child Feeding Counselling: an Integrated Course translated and adapted for the Brazilian context.	Information on BF, feeding HIV-positive mothers, complementary feeding of children 6–24 months, theory of human communication and professional-client interpersonal relationships.	34 sessions (40 h): 8 h of practical sessions (4 in a maternity unit, 2 in a kitchen facility and 2 at a health clinic).	1 director (paediatrician with expertise in these courses) and 11 facilitators (paediatricians, nutritionist, nurses & psychologist) who met pre-requisites.	1) Questionnaire on Profile of each Professional (PPQ) –	Significant improvement only in the practice of obtaining a breastfeeding history. The proportion of individuals whose knowledge of BF skills was higher in the intervention group (89.7%) than in the control group (33.3%) (p < 0.001) across all topics assessed
		A maternity unit, kitchen facility, clinic.						2) Questionnaire on practice of counselling on infant and young child feeding (IFCQ)	
								3) Protocol for observation of clinical consultations (CCOP)	
Blackman et al. (2015) Australia	**Design:** Pre and Post Test study. **Aim:** To measure midwifery students' self-reported abilities in teaching and supervising breastfeeding mothers.	Convenience sample of 95 Bachelor of Midwifery students undertaking topic between 2009-2011. University Setting.	No formal pre-requisite training required.	(a) enhance students' knowledge of maternal and infant nutrition	Human nutrition; Motivate and support; Positioning; attachment; Maintain supply; Removal from breast; keeping baby awake during feeding, assess baby has finished feeding; exclusive breastfeeding.	36 h of tutorials, 108 h of self-directed learning	Not disclosed.	Demographics collected: Age, gender and workplace (hospital or private) Self-reported assessment of knowledge and skills using 4-point Likert scale. 37-item self-report tool designed to estimate self-efficacy	Skills easier post intervention: Determining if baby is getting enough milk; detaching baby from the breast; motivating mother to BF successfully; knowing when the baby has finished feeding, exclusive BF.
				(b)WHO/UNICEF ten steps to successful breastfeeding					Skills harder post topic: BF in public; hold baby comfortably; feed 2-3 hrly; focus on getting through one feed at a time; provide the mother with the rationale for feeding the baby overnight.
				(c) counselling and management for feeding.					There was an overall higher rate in ability but most skills had no significant change in self-efficacy.
Feldman-Winter et al. (2010) USA	**Design** Pre and Post Test study. **Aim:** To evaluate a residency curriculum for improved physician knowledge, practice patterns, and confidence in providing breastfeeding care & whether curriculum was associated with increased breastfeeding rates in women.	417 residents. Residency programmes implemented new curriculum (n = 6), versus those who did not (n = 7) 450 medical charts analysed	All undergoing training to become doctors	The AAP Breastfeeding Residency Curriculum A field-trip design and the Wellstart Lactation Management Self Study Modules integrated into curriculum.	7 major sections: advocacy, community outreach and coordination of care, anatomy and physiology, basic skills, peripartum support, ambulatory management, & cultural competency.	Self-study; discussion questions; didactic lectures; skills workshops; clinical rotation; field trip; presentations; clinical case scenarios; cultural competency cases and hands-on practice.	Two faculty members from each implementation site underwent curriculum-training programme.	1) Knowledge measured by 25 items in the pre-test and 26 items in the post-test	Trained residents more likely to show improvements in knowledge (odds ratio [OR]: 2.8 [95% confidence interval (CI): 1.5–5.0]), practice patterns related to breastfeeding (OR: 2.2 [95% CI: 1.3–3.7]), and confidence (OR: 2.4 [95% CI: 1.4–4.1]) than residents at control sites. Infants at the institutions in which the curriculum was implemented were more likely to breastfeed exclusively 6 months after intervention (OR: 4.1 [95% CI: 1.8–9.7]).
								2) An ordinal scale for residents' confidence.	
								3) Practice patterns of (a) assessment of breastfeeding;	
								(b) counselling about feeding choices; and (c) teaching breastfeeding techniques.	
								BF rates at study initiation and 6 months post study.	
Folker et al. (2018) USA	**Design:** Pre and Post Test study. **Aim:** To determine the effectiveness of the toolkit education by measuring pre- and post-test scores.	2nd yr nursing students (n = 114) self- enrolled in the maternal-newborn nursing 4-week courses. urban community college degree nursing program.	3-part voiceover PowerPoint presentation and reading of role-play activities.	Guided by United States Breastfeeding Committee core competencies and maternal-newborn faculty input.	Self-attachment of the newborn; assisting a new mother with comfortable positioning and latch; use of a double-electric breast pump. An (optional) unfolding case could be accessed anytime on course platform.	Multi-instructional methodology for different learning styles and to foster positive attitudes for BF support. Voice-over PP, role play, classroom-based learning, online case study, demos, and Q&A sessions.	Limited details disclosed. Principle investigator facilitated some training. Content reviewed by 3 doctoral prepared nurse educators with special interest in lactation	Pre and post-test survey design with an intervention and comparison group.	Student learning about breastfeeding was evident from pre-test to post-test scores overall; however, post-test scores remained low.
									Post-test scores were lowest on breastfeeding support skills.
									Since the case study was not made a requirement by every course faculty member, one-third (21/60) of the students in the intervention group did not complete the case study.
Gary et al. (2017) USA	**Design:** Observational study. **Aim:** To evaluate how the US undergraduate medical institution incorporates BF medicine into its curriculum and suggest modifications to improve BF education.	Electronic survey sent to medical students from years 1–4 (response n = 137).	Undergrad Medical Students from years 1–4.	Curriculum used for all 4 years at the USA Liaison Committee on Medical Education accredited undergraduate medical institution.	Basic anatomy and physiology of the; breast; obtaining a BF history, recommending BF over formula feeding, appropriate timing of introduction of solid foods.	Lectures. 4th yr elective: shadow a IBSLC/physicians; attend BF support groups; complete online BF modules; present on BF topic of choice.	Limited information disclosed. Elective field trip support by IBSLC and physicians.	Electronic survey comprised of 15 questions that evaluated knowledge of BF, exposure to BF education, and confidence in counselling about various BF topics.	Most respondents reported they were lacking confidence in counselling families about specific BF topics, contraindications to BF, physiology of lactation, assessing infant latch, establishing & maintaining milk supply & discussing common BF problems.
									Most did not opt for the elective. Medical students were not prepared to counsel women about BF.
Ingram et al. (2011) UK	**Design:** Pre and Post Test study. **Aim:** To evaluate the effects of Baby Friendly Initiative (BFI) community training on BF rates, staff, and mothers in a large Primary Care Trust (PCT).	Invited to take part:	All qualified HCP's. 75% of staff had children and 95% of these BF.	Baby Friendly Initiative seven-point community programme.	Lactation related topics including importance of BF, how lactation works, positioning, attachment, hand expression, recognising and treating conditions/challenges, practical training in skills needed to support successful BF and informed decision making (incl. observing BF, teaching positioning and the attachment, and hand expression).	3-day management course. Three clinical practice opportunities to teach practical skills (2 of which are supervised).	141 Health Visitors (HV) & Nursery Nurses (NN).	Stage 1) 100 HV and 37 NN participated in 3 stage survey (Questionnaires were given immediately before, 1 month after training and 6 months later). Stage 2) 43 HV, NN and managers participated in process evaluation interviews 2–3 months after training. Stage 3) Small survey of mothers (2 months before training, and within six months after HCP training).	**Skills:** Significant increases in skills to give appropriate advice for BM insufficiency, mastitis, and recognising symptoms of poor attachment.
		-All Health Visitors (HV) & Nursery Nurses (NN).							Significant increases in staff attitudes to, knowledge of and self-efficacy with supporting BF, maintained 6 months post training.
		-Managers.							**BF Rates:** Statistically significant increases in any BF and exclusive BF at eight weeks when comparing the rates over 4 years 2006–2009. Babies born in 2009 were 1.57 times more likely to BF and 1.46 times more likely to exclusively BF.
		-Small sample of women.							**Staff Interviews:** Staff supported course. Renewed enthusiasm, improved consistency of advice and confidence levels. Positive feedback from staff motivated managers to encourage attendance at training.
		Community Setting.							**Mothers' survey:** Increase in mothers who BF exclusively. Sample too small for statistical significance
Law et al. (2007) UK	**Design:** Pre and Post Test study.	Midwives on postnatal wards in the four hospitals allocated to intervention (n = 108). Student midwives in control group (n = 27)	All the 108 experimental group participants were registered practicing midwives.	Best Start Breastfeeding (BSB) Trial.	Knowledge about anatomy and physiology of lactation, presentation on correct positioning and attachment, Correct positioning, and attachment.	4 hr workshop in groups of 6–8 staff. Interactive role-play exercises teaching a ‘coaching style’ communication to achieve a ‘hands-off’ support approach.	Midwifery breastfeeding advisers attached to each hospital site.	Biographical questionnaire and modified BeSST at the start of the workshop, BeSST repeated post-intervention.	Significant increases in midwives' knowledge and problem-solving skill level.
	**Aim:** To determine whether a 4-h training programme in ‘hands off’ positioning and attachment support increases midwives' knowledge and problem-solving skills.								
McIntyre et al. (2018) UK	**Design:** Longitudinal Study lasting the length of the students' programme.	22 Midwifery students across 5 NHS Trusts.	All training HCPs.	Baby Friendly Initiative (BFI).	Year 1: Essentials of infant feeding and practicalities of infant feeding. Year 2: Evidence-based practice. Year 3: Challenges in infant feeding with OSCE style workshop. Recorded clinical skills, BF observations and competencies within clinical practice	Lectures, Workshops (OSCE), Clinical practice.	Not disclosed.	A multiple case study approach was used.	**Skills and Knowledge:** Students described the clinical environment as having a predominantly hands-on approach to BF support. Hands-off approach taught to students was challenging as it was not observed during clinical practice.
	**Aim:** A curriculum review of a three-year BSc Midwifery programme.							Questionnaires were answered over 3 years exploring students theoretical and clinical experience. This included skin-to-skin and infant feeding at birth, postnatal infant feeding advice, full breastfeeding observational opportunities and complicated maternal and baby feeding challenges.	Hands-off approach was a step by step process, aided by knowledge of feeding cues, effective communication styles, use of props, embedded by internalisation and daily practice.
								Interviews at all three data collection points were held.	Positive role-modelling changed unevidenced behaviour patterns.
Radoff et al. (2019) USA	**Design:** Pre and Post Test study.	20 obstetrics & gynaecology residents in Boston University.	All medical students.	Learning objectives were conceived through informal conversations with current residents. Reflect ICM and CREOG competencies	Lactogenesis, prenatal, intrapartum and postpartum interventions, hands-on latch assistance, hand expression, use of breast pumps, storage of human milk, and common lactation disorders	3 didactic lectures & 4 h hands-on workshop in a simulation centre.	Local and hospital IBCLCs, a rotating 4th year medical student participating in the breastfeeding elective, midwifery, obs/gynae faculty members.	A voluntary	Significant differences between pre- and post-education results, demonstrating an increase in confidence with breastfeeding knowledge, support, and planned behaviour.
	**Aim:** To describe and evaluate a midwifery-led development of the BF education curriculum for obstetrics and gynaecology residents.		Majority of pre- and post-test respondents had not been exposed to breastfeeding educatio.					15-question survey with a 5-point Likert scale regarding confidence with lactation education and support was administered prior to and after the educational intervention.	Pre-test scores on all aspects of BF hands-on support, counselling, and problem solving were low; whilst confidence with knowledge of the benefits of human milk was high in both pre- and post-tests.
Tender et al. (2014) USA	**Design:** Quasi—experimental study.	39 paediatric residents, assigned to one of three groups. during Well Baby Nursery rotation at community hospital not designated as Baby Friendly.	All medical students, some with prior experience of observing or speaking with breastfeeding mothers.	Questionnaires were formulated using competencies from AAP Breastfeeding Residency	Group 1) shadowed sn IBCLC for a 1-hour session with mothers	Observation of an IBCLC, observation of a prenatal parent breastfeeding class (CLS) and watching a case-based DVD.	IBCLC and Paediatricians.	-Questions from AAP Breastfeeding Residency Curriculum	**Skills:** Residents' in all three groups performed well on the OSCEs with the majority assisting with infant position and latch. Several residents commented that the SP was a valuable teaching asset due to her direct feedback and demonstration of correct position and latch.
								-Knowledge was assessed using a 23-question multiple choice pre-/post-test.	**Confidence**: Resident confidence managing common breastfeeding problems and answering parents' questions about breastfeeding also improved significantly for all 3 groups. Between group difference were not statistically significant.
	**Aim:** To study 3 different time-efficient breastfeeding curricula.			Curriculum	Group 2) watched DVD on BF counselling and example cases Group 3) Observed a 3 hr breastfeeding class given to expectant parents by IBCLC			-Confidence level for addressing parents' BF problems	
								- Resident pre/post self-rating on 5-point scale.	
								-Clinical skills - 1 station OSCE with Standardised Women scenarios.	
Zakarija-Grkovic et al. (2010) Croatia	**Design:** Pre and Post Test study.	HCP's in 5 maternity hospitals between 2007-2009. Midwives, nurses, paediatricians gynaecologists. (n = 424 pre, n = 308 post)	All qualified HCP's.	Baby Friendly Initiative UNICEF/WHO.	15.5 h of theory and 4.5 h of practice relating to BF promotion and support.	UNICEF/WHO 20 h breastfeeding training (2006) (15.5 h theory and 4.5 h of practice relating to breastfeeding promotion and support.	Not disclosed	Anonymous questionnaires	The proportion of HCP who recognised hospital practices that support BF and signs of poor positioning nearly doubled after training. The practice of immediate skin to skin following CS under local anaesthetic was more likely to be supported by younger respondents. Management of mastitis improved significantly. Inappropriate recommendation for partial or complete cessation of BF remained high (47%). Managing insufficient milk supply and management of breastmilk substitutes improved.
	**Aim:** To evaluate knowledge, practices, and attitudes to breastfeeding among Croatian health professionals before and after the (UNICEF/WHO) 20-hour course.		Large proportion of HCP's in the study had children who were breastfed					Questionnaire included 9 items on BF knowledge, 6 items related to BF practice, 17 items relating to BF attitudes (IOWA Feeding Attitudes Scale), and 6 demographic/personal questions.	

ACOG: American College of Obstetrics and Gynaecologists.

AAFP: American Academy of Family Physicians.

AAP: American Academy of Pediatrics.

BBSST: Breastfeeding Best Start Study.

BF: Breastfeeding.

CREOG: Council on Resident Education in Obstetrics and Gynaecology.

ICM: International Confederation of Midwives.

United States of America

WHO: World Health Organisation

### 2.4 Risk of bias assessment

Quasi-experimental studies in the review were assessed using the Cochrane Collaboration Risk of Bias In Non-Randomised Studies of Interventions (ROBINS-I) (Sterne et al., 2016). The observational studies were assessed for risk of bias using criteria based on guidelines for ‘STrengthening the Reporting of OBservational Epidemiological’ (STROBE) studies which Sanderson et al. (2007) reported as incorporating the key principle sources of bias (Philpott et al., 2017). These criteria are selection bias; measurement bias; design specific bias; confounding bias; statistical method bias; and conflict of interest or funding sources (Sanderson et al., 2007). One author (LFP) completed the RoB assessments; these were then independently cross-checked by a second author (RB). Discrepancies were resolved through consensus between the authors.

## 3 Findings

### 3.1 Study selection

**Figure 1:**
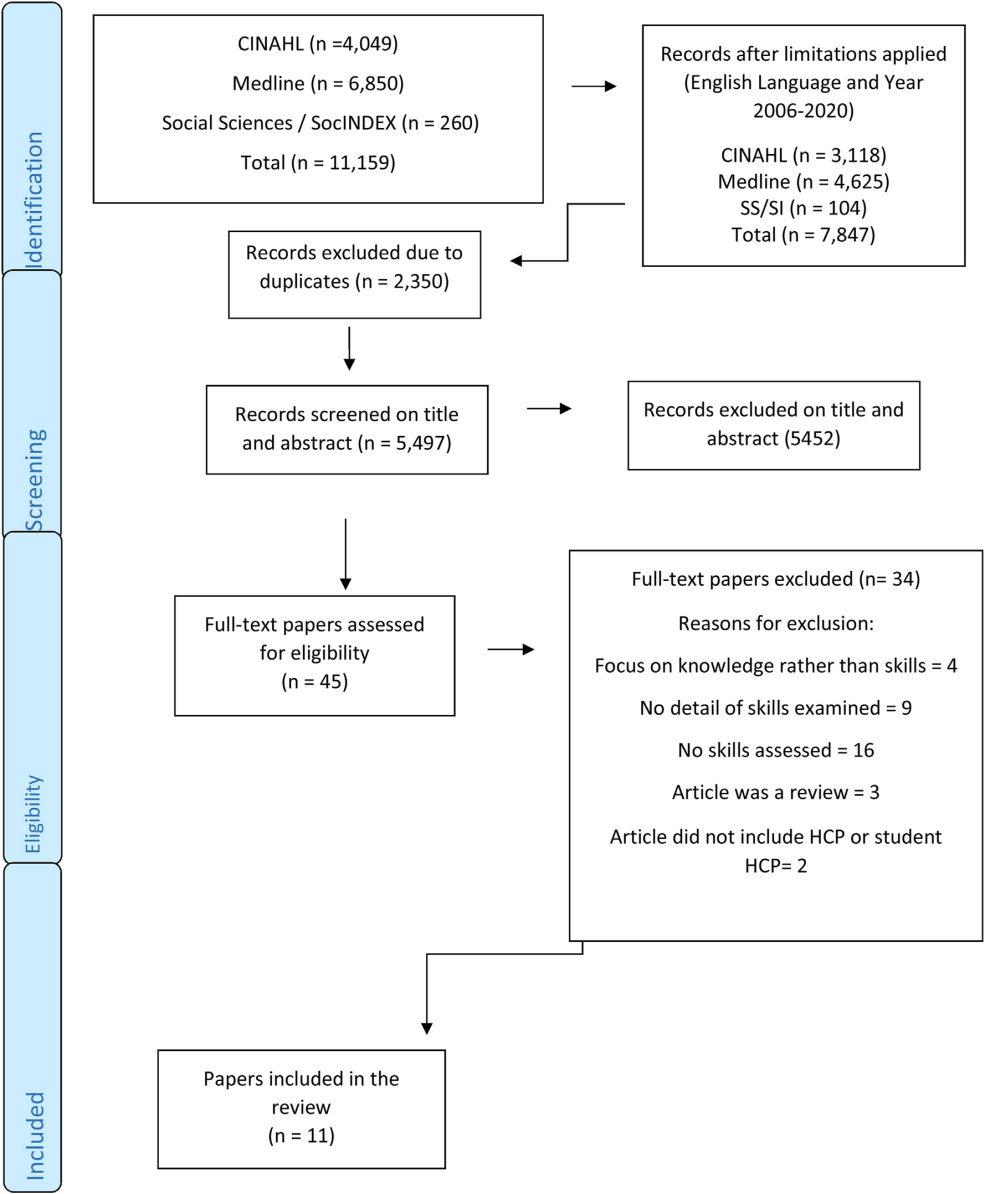
Study selection flow diagram (PRISMA 2020).

The electronic search strategy yielded a total of 7,847 records, and duplicates were deleted (n = 2,350). Titles and abstracts of 5,497 papers were screened and 5,452 papers were excluded, leaving 45 papers for full text review. A total of eleven papers were identified for inclusion. The selection process is presented in Figure 1.

### 3.2 Characteristics of studies

Data extraction of the relevant studies is presented in Table 2. Five studies were conducted in the United States of America (USA) (Feldman-Winter et al., 2010; Folker-Maglaya et al., 2018; Gary et al., 2017; Radoff and Forman, 2019; Tender et al., 2014); three in the United Kingdom (UK) (Ingram et al., 2011; Law et al., 2007; McIntyre and Fraser, 2018); one in Brazil (Bassichetto and Réa, 2008); one in Croatia (Zakarija-Grković and Burmaz, 2010); and one in Australia (Blackman et al., 2015).

Of the eleven studies, eight were pre and post-test designs (Blackman et al., 2015; Feldman-Winter et al., 2010; Folker-Maglaya et al., 2018; Ingram et al., 2011; Law et al., 2007; Radoff and Forman, 2019; Zakarija-Grković and Burmaz, 2010), two were quasi experimental (Bassichetto and Réa, 2008; Tender et al., 2014) and two were observational studies (Gary et al., 2017; McIntyre and Fraser, 2018).

Participants in the studies included: paediatric residents; student midwives; registered midwives; nursing students; health visitors; nursery nurses; managers; service users; nutritionists; care co-ordinators; clinical educators; medical students; obstetrics and gynaecology residents; and paediatric residents. Sample sizes ranged from 20 (Radoff and Forman, 2019) to 424 (Zakarija-Grković and Burmaz, 2010). Participants were recruited from higher education or hospital settings.

Training took place in a classroom, a practical workshop setting, or a clinical setting (including hospital or community). Teaching strategies employed included: didactic lectures; self-study; clinical skills workshops; lectures; presentations; clinical case scenarios; hands-on practice; voice over power point presentations; role play; online case studies; elective placements; clinical observation; simulation workshop; and watching a DVD. Learning opportunities that occurred within a clinical setting were deemed effective strategies to engage participants in practical skills and provide realistic examples of the breastfeeding challenges they were likely to encounter in their profession.

### 3.3 Prerequisites for breastfeeding training

For this paper, prerequisite training is considered and refers to any directed or passive learning that was obtained or recommended prior to taking part in the breastfeeding education programme.

As all eleven of the studies included qualified and student healthcare professionals, it was deemed reasonable to implicitly assume that participants would have some baseline breastfeeding education and training prior to undergoing the enrolling in a breastfeeding education programme. However, based on the pre-test questionnaires in some studies it was evident that healthcare professionals from a variety of disciplines did not possess evidence-based skills to support breastfeeding. One study found that there was a large variation of knowledge amongst midwives. Those that had more experience working on postnatal ward had less post-registration experience and those that had completed some form of specific breastfeeding training had a higher baseline score pre-intervention (Law et al., 2007). Another study, which included medical residents, using a (voluntary) pre-test questionnaires found that most participants had not been exposed to any breastfeeding education previously (Radoff and Forman, 2019). Only two studies took into consideration the number of participants who had breastfed their own children (Ingram et al., 2011; Zakarija-Grković and Burmaz, 2010).

Most of the studies (n = 11) did not state if they recommended or required any prerequisite training. Only one study provided specific instruction in relation to prerequisite training by advising study participants to come prepared for the class by watching a 3-part voiceover PowerPoint and reading the role-play activities (Folker-Maglaya et al., 2018). There was no assessment in this study to determine if participants gained any skills from this activity. Furthermore, the study did not explicitly state whether prerequisite training was mandatory, desirable, recommended, or optional, nor did it specify if they required proof that students had engaged in the prerequisite training.

### 3.4 Educational curriculum

In this paper, Educational Curriculum refers to all academic content used during breastfeeding skills training. Under this heading the following four areas were examined: *‘guiding framework’, ‘content included’, ‘teaching strategy’* and *‘qualifications of educator’.*

#### 3.4.1 Guiding framework

Although teaching strategies differed between the eleven studies, the frameworks used for guiding the breastfeeding skills training were similar. Half of the studies were guided by the WHO/UNICEF Baby Friendly Hospital Initiative and Ten Steps to successful breastfeeding. Three of the USA studies referred to the use of different national curriculums and guidelines to guide their curriculum frameworks, one of which included the American Academy of Pediatrics (AAP) Breastfeeding Curriculum (Tender et al., 2014).

#### 3.4.2 Content included

The breastfeeding skill topics of positioning and latch; problem solving and overcoming common breastfeeding challenges was covered in all eleven studies. However, depending on the study, the educational curriculum placed a different emphasis on some of the breastfeeding skills. For example, two studies emphasised the need for participants to learn a ‘hands off’ approach (Law et al., 2007; McIntyre and Fraser, 2018). The location in which the study took place appeared to impact on the focus of breastfeeding knowledge and skills. For example, one study which was carried out in Brazil, a country with a higher prevalence of HIV, emphasised the need for an integrated breastfeeding course which provides participants with the skills necessary to support HIV-positive mothers to feed their infants (Bassichetto and Réa, 2008).

Additional skills taught and assessed included: interpersonal skills (Bassichetto and Réa, 2008; Blackman et al., 2015; Law et al., 2007; Tender et al., 2014) obtaining a breastfeeding history (Bassichetto and Réa, 2008; Gary et al., 2017); assessment of adequate milk supply (Tender et al., 2014); maintenance of supply (Blackman et al., 2015; Gary et al., 2017), removal of infant from breast (Blackman et al., 2015); maintenance of an awake state during feeding (Blackman et al., 2015); recognition of conclusion of feeding (Blackman et al., 2015); introduction of solid foods (Bassichetto and Réa, 2008; Gary et al., 2017); usage of breastfeeding support devices such as a breast pump, nipple shields and nursing supplementation (Folker-Maglaya et al., 2018; Radoff and Forman, 2019).

#### 3.4.3 Teaching strategy

A diverse range of teaching strategies were used across the eleven studies. They included classroom taught tutorials; self-directed learning; didactic lectures, observation of breastfeeding support provided by a qualified member of staff; simulation sessions, voluntary electives, and training workshops (Law et al., 2007); simulation scenarios; open-discussion lectures; web-based learning; and watching an interactive video (Tender et al., 2014).

Only one study referred to the use of reflective practice in the learning process (McIntyre and Fraser, 2018). In documenting a student's reflection of a clinical scenario, McIntyre and Fraser (2018) identified the prevailing use of ‘hands-on’ breastfeeding support by qualified midwives and the challenges experienced by more junior members of the healthcare team face in changing such practices.

Teaching strategies that incorporated an observational or practical element had better outcomes than those that used classroom-based teaching strategies in isolation. One study which assessed an undergraduate medical curriculum at the US medical institution found that most medical students did not achieve the recommended competencies at the end of their four years of medical training. Although there was an elective offered to student, which allowed extensive practical exposure to breastfeeding, it had little impact as it was only accessed by students who voluntarily choose to take this extra course (Gary et al., 2017).

Folker-Maglaya et al. (2018) adopted a multi-instructional methodology to meet the needs of different learning styles. Voice over Power Point; role play; classroom-based learning; an online case study; demonstrations; and question and answer sessions were used as teaching methods. However, based on pre- and post-test scores learning in relation to breastfeeding skills remained low. Researchers in this study, emphasised the need for students to have the opportunity to practice breastfeeding skills either in the clinical setting or in a simulated laboratory (Folker-Maglaya et al., 2018). Similarly, in another study (Blackman et al., 2015), it was concluded that alongside theory-based learning, students require other avenues to learn breastfeeding skills, such as greater exposure to breastfeeding women and lactation services.

Radoff et al.’s (2019) teaching strategy involved a total of three 3-hour didactic lectures, followed by a 4-hour hands-on workshop in a simulation centre. Likewise, two of the three teaching sessions in Tender et al.’s (2014) involved students observing breastfeeding counselling and classes in a clinical setting. Tender et al. (2014) choose these teaching methods as they were deemed to be time efficient and could be easily integrated into paediatric residents' course timetables.

In Radoff et al.’s (2019) study pre- and post-test results demonstrated higher rates of knowledge, planned behaviour, support skills and confidence. Unlike previous breastfeeding education delivered to study participants, which was geared towards teaching students the theoretical benefits of breastfeeding, this study focused on improving students' knowledge of and confidence in providing practical breastfeeding support. Post intervention one student reported that they now ‘actively think’ of practical ways in which they can support breastfeeding in hospital settings. Similar results were found in other studies which used practical skills-based training interventions when teaching healthcare students and qualified healthcare professionals (Feldman-Winter et al., 2010; Ingram et al., 2011; Law et al., 2007; McIntyre and Fraser, 2018; Tender et al., 2014; Zakarija-Grković and Burmaz, 2010).

#### 3.4.4 Qualifications of educator

Educational interventions were delivered by different professionals with knowledge and experience in the field of breastfeeding. Educators included university staff (midwifery, obstetrics and gynaecology faculty members), community and hospital based IBCLCs, and healthcare professionals with a special interest in lactation (e.g. Midwife, Paediatrician, Nutritionists, Nurses, Psychologists, Medical Student, Health Visitors and Nursery Nurses). Some studies stated that the educators undertook (Feldman-Winter et al., 2010) or were required to have undertaken (Bassichetto and Réa, 2008) training prior to the educational intervention, whilst most of the studies did not disclose if the educators, undertook training. One study found that positive role-modelling from a skilled mentor was very powerful in changing unevidenced behaviour amongst student healthcare professionals (McIntyre and Fraser, 2018). A quarter of studies (n = 3) did not report on the qualifications of the facilitator for their educational intervention (Blackman et al., 2015; McIntyre and Fraser, 2018; Zakarija-Grković and Burmaz, 2010).

Several studies used educators from more than one discipline. For example, in Radoff et al.’s (2019) study each lecture was facilitated by a different educator. The first lecture was given by two International Board-Certified Lactation Consultants (IBCLC); the second lecture was taught by a rotating medical student who was participating in the medical schools midwifery led breastfeeding medicine elective clerkship; and the third lecture was presented as a panel discussion with faculty members from midwifery, obstetrics, and gynaecology. Study authors attributed the success of the educational intervention to the pre-existing strong relationships amongst the midwifery and medical faculty at the university, and between the university faculties and the in-hospital and community lactation support teams. In addition to attending three didactic lectures students were invited to participate in a practical session, whereby women and their infants had volunteered to support student learning in a clinical skill setting. Students had five stations to complete where they had the opportunity to deliver breastfeeding counselling and advice to women under direct supervision.

#### 3.4.5 Assessment of learning

All studies reviewed recorded some measure for assessing learning of breastfeeding skills. Although not all studies succeeded in improving breastfeeding skills based on their assessments, all studies acknowledged the need for educational curricula that improve the practical breastfeeding skills of healthcare professionals.

Questionnaires were the main method of data collection used in the eleven studies and there was no evidence of standardisation across studies. In all questionnaires/surveys, healthcare professionals’ skills or self-efficacy in relation to their ability to provide breastfeeding skills were assessed. Most studies asked participants to voluntarily complete a questionnaire/survey, some of which incorporated a 5-point Likert scale. In some studies, the content of the questionnaires/surveys was based on competencies from established curriculum or national standards, for example Tender et al. (2014) used questions from the validated AAP Breastfeeding Residency Curriculum.

In conjunction with questionnaires/surveys some studies assessed learning using interviews or clinical skills assessments. Of the eleven, two studies included interviews with participants who had engaged in the training (Ingram et al., 2011; McIntyre and Fraser, 2018). Interestingly, findings from one study indicated that receiving positive feedback from staff helped motivate hospital managers to encourage and provide time for healthcare staff to attend training (Ingram et al., 2011).

Out of the eleven studies, only two studies objectively assessed clinical skills (Bassichetto and Réa, 2008; Tender et al., 2014). Tender et al. (2014) did so, through a one station objective structured clinical examination (OSCE), which consisted of a standardised person scenario. The participants were scored with a twenty-two-item scoring form that was divided into four sections: interpersonal skills, maternal history taking, assessment of milk supply, clinical assessment, and counselling skills (Tender et al., 2014).

In addition to assessing healthcare professionals' skills and experiences, two studies assessed women's experiences of receiving breastfeeding counselling (Ingram et al., 2011) or compared breastfeeding rates pre- and post-the intervention (Feldman-Winter et al., 2010). One study found that not only did participants develop individual skills but that infants at the institutions in which the curriculum was implemented were more likely to breastfeed exclusively 6 months after intervention (Feldman-Winter et al., 2010).

### 3.5 Training outcome

As discussed in the Teaching Strategy section of this paper, studies that included an observational or experiential opportunity had better outcomes when compared to those that were classroom-based (Ingram et al., 2011; Radoff and Forman, 2019; Tender et al., 2014; Zakarija-Grković and Burmaz, 2010). In these educational interventions' participants learned a variety of practical skills to support breastfeeding including how to recognise a good latch and respond to common breastfeeding challenges. ‘Hands off’ support was a skill taught in two of the studies examined (Law et al., 2007; McIntyre and Fraser, 2018). To achieve ‘hands off’ breastfeeding support, both studies identified effective communication as a necessary skill and taught participants how to communicate using a coaching style approach.

Finally, few studies took into consideration the wider systemic and cultural influences that may impact on the short- and long-term success of the educational intervention. McIntyre et al. (2018) acknowledged that the practice area and mentors available to students are very influential and have the power to reaffirm positive breastfeeding practices.

### 3.6 Risk of bias

For the quasi-experimental studies (Table 3), only Tender et al. (2014) had ‘low’ RoB due to confounding variables, while 5 studies had high RoB for selection due to convenience sampling and/or self-selection. For domain 4, bias due to deviations from intended interventions, all the studies were deemed ‘low’ RoB. All studies except for Feldman-Winter et al. (2010) were considered as ‘low’ RoB for domain 7 which is related to bias in selection of reported result. The majority of studies (n = 7) provided no information on missing data (Bassichetto and Réa, 2008; Blackman et al., 2015; Feldman-Winter et al., 2010; Folker-Maglaya et al., 2018; Ingram et al., 2011; Law et al., 2007; Zakarija-Grković and Burmaz, 2010). Finally in domain 6, bias in measurement of outcomes, 4 studies were ‘high’ RoB (Bassichetto and Réa, 2008; Folker-Maglaya et al., 2018; Ingram et al., 2011; Radoff and Forman, 2019); 4 studies were ‘low’ RoB (Feldman-Winter et al., 2010; Law et al., 2007; Tender et al., 2014; Zakarija-Grković and Burmaz, 2010) and 1 study was moderate RoB (Blackman et al., 2015).

**Table 3:** Cochrane Collaboration's ROBINS-I tool for assessing risk of bias in non-randomised studies of interventions (Sterne et al., 2016).

Study	D1	D2	D3	D4	D5	D6	D7	Overall
Bassichetto et al. (2008)								
Blackman et al. (2015)								
Feldman-Winter et al. (2010)								
Folker et al. (2018)								
Ingram et al. (2011)								
Law et al. (2007)								
Radoff et al. (2019)								
Tender et al. (2014)								
Zakarija-Grkovic et al. (2010)								

Domains: D1: Bias due to confounding. D2: Bias due to selection of participants. D3: Bias in classification of interventions. D4: Bias due to deviations from intended interventions. D5: Bias due to missing data. D6: Bias in measurement of outcomes. D7: Bias in selection of reported result.

Judgement:  Serious,  Low,  Moderate,  No Information.

For the observation studies (Table 4), risk of selection bias was high in both studies and was related to convenience sampling and/or self-selection (Gary et al., 2017; McIntyre and Fraser, 2018). Risk of bias regarding the use of appropriate measures was high in one study as the authors did not provide information regarding the validity or psychometric robustness of the instrument used (Gary et al., 2017). In the other study a judgment of unclear was rendered as the authors provided no information about the measurement tool that they used (McIntyre and Fraser, 2018). In both studies an unclear risk of design specific bias was made as attrition rates and/or recall bias was not reported. Risk of bias in relation to control of confounders was high in both studies, with an unclear judgment as the use of statistics for primary analysis of effect was not reported. Conflict of interest was low in 1 study (Gary et al., 2017), and unclear in the other study as the authors did not report on the conflict of interest or funding (McIntyre and Fraser, 2018). Therefore, due to serious high risk of bias in all but one of the of studies included in this review, the findings should be interpreted with caution.

**Table 4:** Criteria for assessing quality and susceptibility to bias for observational studies (Adapted from Sanderson et al., 2007).

Author, Year	Selection bias, Sampling source and methods, with inclusion/exclusion criteria	Measurement Bias, Exposure and/or Outcome measurement	Design Specific Bias, Attrition, Recall	Confounding Bias	Statistical Method Bias, Primary analysis of effect	Conflict of Interest or funding source
Gary et al. (2017)	H	H	UC	H	UC	L
McIntyre et al. (2018)	H	UC	UC	H	UC	UC

H=High; L = Low; UC = Unclear.

## 4 Discussion

This study was not limited to education for one specific professional group, but reviewed research papers which examined breastfeeding skills-based training to any healthcare professional, including student healthcare professionals, midwives, nurses, and medical doctors. All eleven studies identified current inadequacies in the breastfeeding skills of their professional groups, and thus purported to use their study as a way to document the effect of implementing a skills-based training in order to change breastfeeding practices. The skills-based training used was sometimes implemented alongside educational interventions to improve breastfeeding knowledge and/or confidence of individual healthcare professionals.

No studies aimed to address wider systemic changes to a healthcare service. A large study, which was not included in the review as it did not contain enough information regarding research design, took a multifaceted approach (Bohling-Smith and Peters, 2011). Rather than delivering education to healthcare professionals in isolation, they also put in place a plan, using WHO/UNICEF Baby Friendly Hospital Guidelines, to address cultural breastfeeding practices within a hospital. Alongside 18 h training for nurses, physicians were required to revise breastfeeding policies and were invited to an attend an annual breastfeeding training; whilst a goal was set that one hundred per cent of care coordinators and clinical educators would undergo training to become lactation consultants. As a result, this 5-year study addressed cultural practices which interfered with successful breastfeeding and developed evidence based breastfeeding skills amongst multi-disciplinary teams. Training outcomes from this study demonstrate the need for breastfeeding education to occur in the context of wider systemic changes (Bohling-Smith and Peters, 2011a).

This review examined the use of prerequisite training which has been found to support participants’ orientation and reduce the amount of background material to cover during classroom trainings. Mayer (1975) identified a three-step model of learning which identifies prerequisite learning as a fundamental basis to the learning process. New information can only be connected to existing knowledge if the knowledge to support the new information exists within the learner. Thus, Mayer found that for meaningful learning to occur, relevant prerequisite concepts must be made available to the learner (Mayer, 1975).

Despite this, most of the studies did not have recommended or required prerequisite training. The reason for a lack of prerequisite trainings in the studies reviewed remains unknown, thus one can only speculate why prerequisite trainings were not used. A possible reason is that the researchers assumed that as qualified and student healthcare professionals, that the participants had some pre-existing level of breastfeeding skills and knowledge. However, as pre-test questionnaires undertaken in this review have demonstrated, healthcare professionals’ skills and knowledge of breastfeeding can vary greatly.

Therefore, it would be reasonable to suggest that prerequisite training may have been of benefit and a way of attempting to provide a basic level of breastfeeding knowledge prior to attending a breastfeeding skills session. An example of prerequisite training in the case of a breastfeeding skills-based training might be, that prior to the participant learning the skills to support a woman with their infant's latch, that students are first required to complete a web-based learning, which enables them to understand the anatomical structures and physiological processes of milk production and excretion.

To guide the framework and models, the eleven studies used a variety of widely recognised resources, including the WHO/UNICEF Baby Friendly Hospital Initiative. Doing so, enabled educators to create trainings with a set of already identified competencies and practice changes needed to improve breastfeeding skills. Frameworks have been found to be desirable in developing a curriculum as they underpin educational content to inform teaching strategies and meet the learning outcomes. For example, one study based in the USA, referred to using the AAP Breastfeeding Curriculum. Doing so, enabled them to create a clinical scoring form to assess medical residents learning outcomes (Tender et al., 2014).

Many similarities existed between the content of the skills-based trainings reviewed, with all eleven studies teaching positioning and latch and problem solving. However, the focus of the skills-based training varied, with two UK studies emphasising the need for a ‘hands-off’ approach to breastfeeding support. Law et al. (2007) found that a ‘hands-off’ approach was associated with significant improvements in midwives' knowledge and problem-solving skill level. However, McIntyre et al. (2018) identified that teaching a skill such as ‘hands-off- support to Midwifery students, can be challenging when the clinical environment does not predominantly practice such a skill. For example, in this case, midwifery students were learning ‘hands-off’ breastfeeding skills in the training, but then being role modelled ‘hands-on’ skills in clinical practice (McIntyre and Fraser, 2018). For these midwifery students this study found that positive role modelling from a more senior member of the midwifery team (e.g. a mentor) brought about changes in unevidenced behaviours, such as using a ‘hands-on’ approach. Developing effective communication skills amongst participants enabled them to develop their competence in describing a skill process to women (Law et al., 2007; McIntyre and Fraser, 2018).

In terms of teaching strategies, approaches that had an observational or practical element were found to have better outcomes then those studies which used classroom-based teaching strategies in isolation. In one study the interaction with ‘real-life’ families was thought to aid students breastfeeding skills, however it was not without its challenges as authors stated it was difficult to recruit mother-infants dyads that were in the early stages of breastfeeding, and thus many of the breastfeeding dyads had already established breastfeeding and therefore were not experiencing the same types of breastfeeding challenges that one might experience if working within a maternity hospital (Radoff and Forman, 2019). Nonetheless, it is evident that breastfeeding women have the potential to play an important role in contributing to the skills based breastfeeding education of healthcare professionals. No other study involved mother-infant dyads in this way. Only one study referred to the use of reflective practice in the learning process a teaching strategy (McIntyre and Fraser, 2018), which is recommended by Dykes (2006) to challenge pre-existing attitudes and behaviours relating to breastfeeding.

Although ‘real life’ observation and practice may be deemed an effective method of learning, the reality of providing these opportunities to participants is lessened by the restrictions brought about by the Covid-19 pandemic. Interestingly, where learning could not take place in a clinical setting, a simulated laboratory was seen to also be effective (Radoff and Forman, 2019). Breaking down the skills necessary for delivering breastfeeding support in this way and allowing participants to practice with one another or in a clinical skills environment, contrasts with a more traditional form of breastfeeding education, whereby healthcare professionals were provided with theoretical information about breastfeeding but not necessarily taught how to acquire breastfeeding skills.

All studies reviewed were undertaken prior to the Covid-19 pandemic, which might explain the low use of e-learning programmes, both in terms of theoretical learning and skills-based simulation. As a result of Covid-19 restrictions, e-learning platforms are now the desired method of learning across many university and healthcare settings. A study conducted in 2018 found that although e-learning programmes provide a flexible teaching method, they do not exceed face to face person simulation. In order to use e-learning programmes effectively this study suggests the need for such programmes to be implemented in conjunction with traditional teaching methods (McDonald et al., 2018).

The qualifications of educators varied, with professionals coming from a range of backgrounds, including academic, community and hospital-based settings. A few of the studies recruited educators from more than one discipline. In one study, the authors attributed the success of the training to pre-existing strong relationships that existed amongst the trainers, in this case the midwifery and medical faculty at a University (Radoff and Forman, 2019). Although, none of the studies recruited breastfeeding parents as a formal educator, one study did invite students to attend a session with mother-infant dyads where they could discuss with the woman her experience of breastfeeding. It is assumed that this sharing of skills and knowledge between the breastfeeding person and the professional occurred informally for those participants who had the opportunity to learn within a clinical setting.

All studies used questionnaires to assess learning outcomes. Some studies used modified questionnaires (Law et al., 2007) or based their questionnaires on already established curriculum (Pries et al., 2016). Questionnaires used various scales for measuring participants' skills, one which was commonly used was the Likert Scale. Questionnaires are a useful research instrument as they enable the research to collect large amounts of quantitative data in a relatively short period (Parahoo, 2008). However, results need to be interpreted with caution as the answers given may not always be an accurate reflection of one's behaviour, in this case their ability to provide breastfeeding skills. Thus, incorporating questionnaires with observational methods enables the researcher to check if participants have been able to integrate their theoretical knowledge into practice and whether what they say they are doing is true (Mcilfatrick, 2008).

Whilst most studies used a pre- and post-test questionnaire to assess learning, some studies relied on observation-based assessments in conjunction with questionnaires (Bassichetto and Réa, 2008; Feldman-Winter et al., 2010; Tender et al., 2014). A study which used an OSCE station developed a clinical scoring form from the AAP Breastfeeding Residency Curriculum OSCE Performance Assessment (Tender et al., 2014). Another study observed a breastfeeding clinical consultation recording the presence and frequency of desired actions (Bassichetto and Réa, 2008).

Interviews were also found to be an effective method of gathering data relating to learning outcomes. Data captured in interviews revealed the positive effect the skills-based training had on changing practices and delivering consistent advice (Ingram et al., 2011). As stated by Jackson et al. (2008) carefully planned interviews are powerful research tools within healthcare research, as they capture the more subtle details of a person experience.

One study measured outcomes using a small survey amongst mothers, two months before the healthcare professional training and six months after the training (Ingram et al., 2011). Another study recorded breastfeeding rates using medical charts at the time point of training initiation and six months post (Feldman-Winter et al., 2010). Another study, which was not included in the review, also used hospital records to assess learning outcomes. In this study, nurses were asked to document exclusive breastfeeding practices in more detail than Feldman-Winter et al.’s (2010) study. Observation of one feed per shift; one to one lactation education and support; discussions of community-based breastfeeding resources; pacifier use and education; supplementation; skin to skin; and hand expression were documented. This interesting and unique approach enabled researchers to capture more nuanced changes to exclusive breastfeeding practices and later track changes in practices over an extended period of five years (Bohling-Smith and Peters, 2011).

Measuring outcomes using person experience surveys (Ingram et al., 2011) and pre-and post-breastfeeding rates (Feldman-Winter et al., 2010) provides some evidence that skills-based educational interventions can lead to outputs that extend far beyond individual skills of healthcare professionals, by having the potential to impact on population breastfeeding rates and improve service user experiences overtime. Methods of assessment which track changes over longer time points and demonstrate measurable outcomes in terms of breastfeeding rates are desirable in terms of Key Performance Indicators (KPI). Obtaining measurable outcomes, such as KPIs has been shown to improve safety and quality of care to women (Health Information and Quality Authority, 2013). Furthermore, such measurable outcomes enable health services to justify the allocation of the resources (Health Information and Quality Authority, 2013) and funding required to deliver educational programmes, in this case breastfeeding skills-based trainings.

In the review, risk of bias was assessed based on study design-specific principles and conduct rather than quality of reporting of methods and results. All but one of the of studies included had a serious or high risk of bias, therefore, the findings should be interpreted with caution. Furthermore, the studies included were heterogeneous, specifically in relation to aims, interventions and educational curricula.

Finally, it is important to acknowledge that healthcare professional's knowledge and attitude towards breastfeeding exists in the context of their own personal experiences and the wider socio-political landscape (Dykes, 2006). Thus, in order to bring about long term, sustainable outcomes, breastfeeding skills-based training for healthcare professionals needs to coincide with wider cultural changes within healthcare settings. The process of achieving positive breastfeeding cultures is twofold, as it then creates an ‘in-house’ learning environment, where future students and staff are supported to learn evidenced based breastfeeding skills from colleagues in the form of role modelling.

### 4.1 Strengths and limitations

A systematic review protocol was developed to guide this review but was not registered and is an acknowledged limitation. Various guidelines, frameworks, and standardised checklists guided this systematic review including STROBE (Von Elm et al., 2007), the Cochrane Collaboration Risk of Bias tool; and the ROBINS-I (Sterne et al., 2016).

### 4.2 Implications for future research and practice

Based on the results of this review, we recommend that an interdisciplinary approach be used in the facilitation of breastfeeding skills-based training. Strong, respectful relationships between disciplines and faculties need to be developed, in areas where they may not pre-exist. All educational interventions should incorporate either a practical and/or observational element alongside theoretical learning. Taking a multi model approach to educational interventions and assessments provides participants with an array of learning opportunities, whilst mutually reinforcing breastfeeding knowledge and skills in a variety of clinical and academic settings. Furthermore, using multiple forms of assessment supports different learning styles and facilitates long-term measurement of training outcomes.

Greater consideration needs to be given to how both student and qualified healthcare professionals consolidate their knowledge following trainings. Furthermore, the retention and implementation of theoretical knowledge and breastfeeding skills needs to be assessed in more detail to establish long term outcomes.

## 5 Conclusion

This review highlighted the variations in teaching, assessment strategies and educator qualifications that exist between breastfeeding skills interventions, with observational or experiential opportunity having better outcomes compared to classroom-based interventions.

The findings have been interpreted cognisant of the serious/high risk of bias identified in all but one of the included studies and reported accordingly. Nevertheless, this review can be used to inform future development of skills-based training for students and qualified healthcare professionals, to improve breastfeeding support for families. Educational interventions, delivered by knowledgeable multidisciplinary facilitators, that incorporated practical and/or observational element(s) with theoretical learning were relatively successful at achieving desired learning outcomes.

With growing awareness of the benefits of breastfeeding and the increased recognition of the importance of continued professional development, breastfeeding educational interventions need to take a multi-model approach, that enables healthcare professionals to challenge pre-existing attitudes relating to breastfeeding; gain up to date skills-based competencies and adopt evidence-based practices when supporting breastfeeding families.

Innovative ways of capturing learning and measurable service outcomes, over extended time periods, needs to be considered to ensure the necessary time, resources and funding is provided to breastfeeding education for healthcare professionals.

Finally, such training for healthcare professionals needs to coincide with initiatives to improve wider cultural changes which support evidence-based breastfeeding practices within healthcare settings.

## Declarations

### Author contribution statement

Helen Mulcahy, Patricia Leahy-Warren: conceived and designed the experiments; analyzed and interpreted the data; wrote the paper.

Lloyd Frank Philpott: performed the experiments; analyzed and interpreted the data; contributed reagents, materials, analysis tools or data.

Roisin Bradley: performed the experiments; contributed reagents, materials, analysis tools or data; wrote the paper.

Michelle O'Driscoll: performed the experiments; analyzed and interpreted the data; wrote the paper.

### Funding statement

Funded by Health Service Executive, Ireland.

This work was supported by Health Service Executive, Ireland.

### Data availability statement

Data included in article/supp. material/referenced in article.

### Declaration of interest’s statement

The authors declare no conflict of interest.

### Additional information

No additional information is available for this paper.
